# A Signature of Nine lncRNA Methylated Genes Predicts Survival in Patients With Glioma

**DOI:** 10.3389/fonc.2021.646409

**Published:** 2021-03-22

**Authors:** Meng Cheng, Libo Sun, Kebing Huang, Xiaoyu Yue, Jie Chen, Zhengwei Zhang, Bing Zhao, Erbao Bian

**Affiliations:** ^1^Department of Neurosurgery, The Second Affiliated Hospital of Anhui Medical University, Hefei, China; ^2^Cerebral Vascular Disease Research Center, Anhui Medical University, Hefei, China

**Keywords:** glioma, long non-coding RNA, methylation, epigenetics, prognosis

## Abstract

Glioma is one of the most common malignant tumors of the central nervous system, and its prognosis is extremely poor. Aberrant methylation of lncRNA promoter region is significantly associated with the prognosis of glioma patients. In this study, we investigated the potential impact of methylation of lncRNA promoter region in glioma patients to establish a signature of nine lncRNA methylated genes for determining glioma patient prognosis. Methylation data and clinical follow-up data were obtained from The Cancer Genome Atlas (TCGA). The multistep screening strategy identified nine lncRNA methylated genes that were significantly associated with the overall survival (OS) of glioma patients. Subsequently, we constructed a risk signature that containing nine lncRNA methylated genes. The risk signature successfully divided the glioma patients into high-risk and low-risk groups. Compared with the low-risk group, the high-risk group had a worse prognosis, higher glioma grade, and older age. Furthermore, we identified two lncRNAs termed PCBP1-AS1 and LINC02875 that may be involved in the malignant progression of glioma cells by using the TCGA database. Loss-of-function assays confirmed that knockdown of PCBP1-AS1 and LINC02875 inhibited the proliferation, migration, and invasion of glioma cells. Therefore, the nine lncRNA methylated genes signature may provide a novel predictor and therapeutic target for glioma patients.

## Introduction

Glioma is the most common primary malignant tumor of the central nervous system. Moreover, glioma has a high recurrence rate, high mortality rate, and low cure rate (1). The World Health Organization (WHO) classifies glioma cells into four types according to their morphological characteristics and prognosis (2). Among the all glioma types, glioblastoma (GBM), a grade IV glioma, is the most common and aggressive intracranial tumor (3). Currently, treatment relies merely on maximum surgical resection and radiation and chemotherapy (4). As a result of gliomas' heterogeneity, their clinical prognoses vary widely with similar treatment in the same grade of glioma (5). Therefore, based on the current classification of glioma grade, it is no longer possible to accurately predict the survival and prognosis of patients. Hopefully, exploration of new molecular biomarkers related to the pathogenesis of glioma may benefit the diagnosis and treatment of glioma with different grades. Molecular studies could provide new biomarkers for the heterogeneity of glioma cells, predict the treatment sensitivity and survival time of different patients, and enable patients to obtain treatment benefits and maximize survival time. Therefore, it is necessary to find a new classification method to classify glioma and predict the prognosis of patients based on the original histological classification system.

LncRNA is a type of RNA that is longer than 200 nucleotides and lacks protein-coding ability (6). Nevertheless, lncRNAs still play an extremely broad role in important life activities of organisms, including epigenetic modification of genes, splicing and editing of RNA, regulation of miRNA, and folding of protein molecules (7, 8). Aberrant expression of lncRNAs has been observed in human cancers, where they can act as either tumor suppressor genes or oncogenes, depending on the environment in which tumor cells are located (9, 10). Multiple studies have shown that the dysregulation of lncRNAs is extensively involved in the malignant progression of human cancers (11, 12). In addition, lncRNAs also play a crucial role in the occurrence and development of glioma. Previous studies have shown demonstrated lncRNAs are involved in cell cycle, epithelial to mesenchymal transformation, DNA repair, angiogenesis, and other molecular mechanisms related to glioma growth (13, 14). In recent years, the biological function and carcinogenic mechanism of lncRNAs in glioma have been widely explored, and it has become a potential therapeutic and diagnostic target for glioma patients. Significantly, our research group has achieved advances in lncRNAs research in the field of glioma. For example, we determined that HOTAIR (15), MEG3 (16), and lncRNA-ATB (17) play a significant role in the malignant progression of glioma.

DNA methylation is a form of chemical modification of DNA that can cause epigenetic changes without altering the DNA sequence (18). It can shut down the activity of specific genes, while demethylation induces gene reactivation and expression. Epigenetic modification of DNA methylation has been reported in a large number of studies on glioma. Numerous cell cycle control genes, DNA damage repair genes, and tumor invasion-related genes are methylated in their promoter regions, which leads to changes in the biological functions of glioma cells (19–21). Studies have shown that the IDH1/2 mutations is closely associated with DNA methylation in gliomas genomes (22). IDH1/2 mutations are common in glioma cells and can completely change the DNA methylation landscape (23). At present, the methylation of lncRNA promoter region has been added to research efforts, and it can affect the biological function of tumors through epigenetics (24, 25). Changes in the methylation level in the promoter or enhancer region of lncRNAs, especially the CpG sites, may significantly alter the epigenetic modification of lncRNA, resulting in the malignant progression of tumor cells (26). A study reported that the hypomethylation of the promoter region of lncRNA SNHG12 led to the upregulation of SNHG12 expression in gliomas, which led to the malignant progression of gliomas (27). Increasing evidence suggests that methylated lncRNA may be a potential prognostic biomarker (24, 28). Due to the heterogeneity of glioma, the study of a single methylated lncRNA biomarker can no longer accurately reflect the characteristics of glioma and predict the prognosis of patients. We hypothesized that the prognostic value could be significantly amended by integrating multiple methylated lncRNA biomarkers into a single model.

In this study, we analyzed the genome-wide methylation profile and the clinical follow-up data of glioma patients through The Cancer Genome Atlas (TCGA, https://cancergenome.nih.gov/) database. The multistep screening strategy identified nine lncRNA methylated genes that were associated with prognosis in glioma patients. In addition, a risk signature that containing these lncRNAs was established. We then investigated the potential biological function of the signature in glioma. The functional experiments were performed in glioma cell lines to verify the risk signature. Finally, our research demonstrated the prognostic value of the nine lncRNA methylated genes signature and provided promising prognostic biomarkers for glioma patients.

## Methods

### Data Downloading and Pre-processing

**Figure 1:**
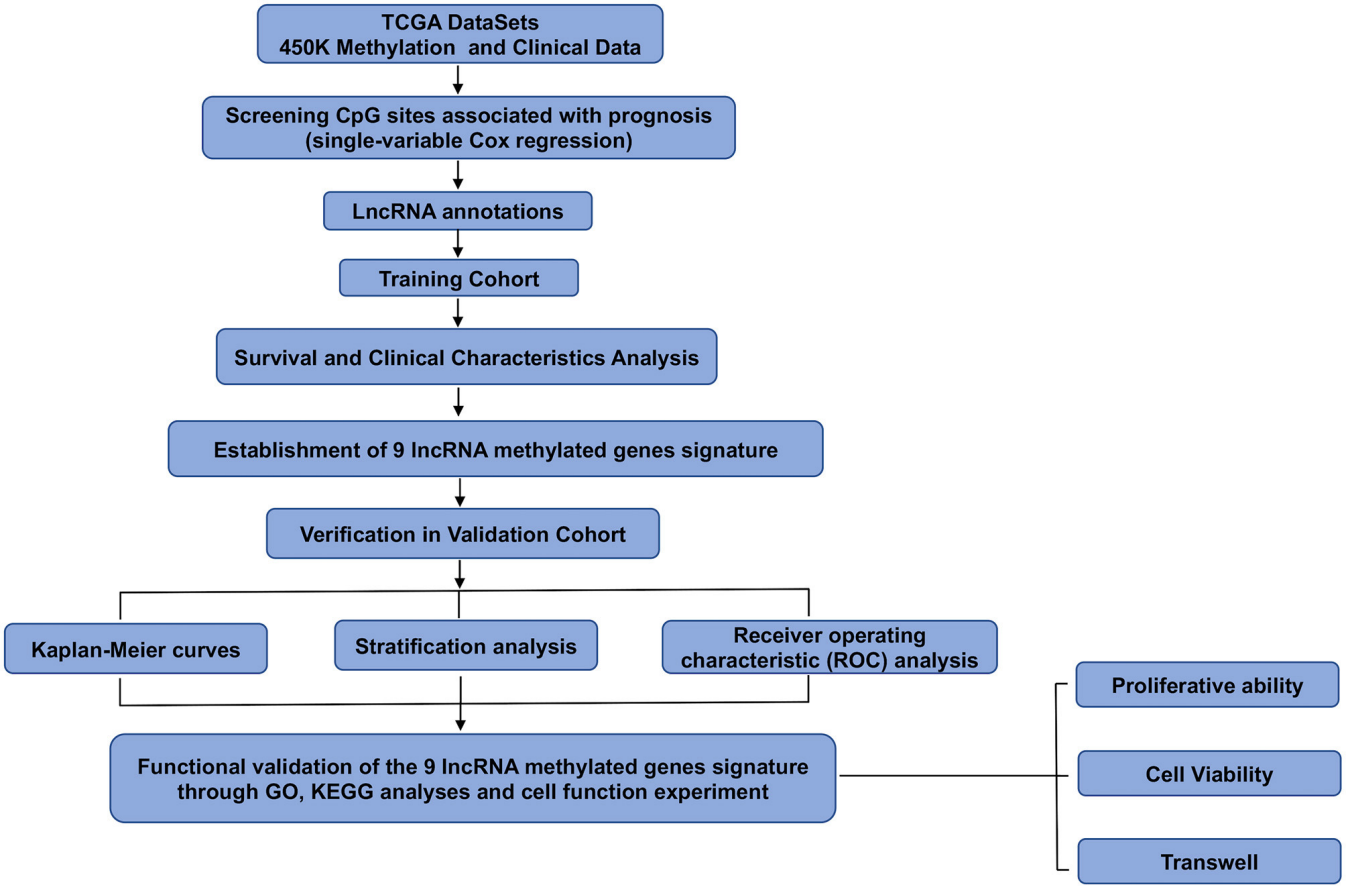
Flowchart of this study.

Data on 450K methylation and the clinicopathological characteristics of glioma patients were downloaded from TCGA. The number of methylation 450K samples from glioma patients was 675, and the number of clinical follow-up cases was 649. The β-values were used to represent the methylation level of each probe, ranging from 0 to 1, representing unmethylated to fully methylated. We used a univariate Cox model to select the main prognosis-related CpG sites and set *p* < 0.05 as the significance threshold. We only included the CpG sites from patients with available survival information. Then, we obtained CpG sites related to patient prognosis. Finally, the total samples were randomly divided into the training cohort (*n* = 325) and the validation cohort (*n* = 324). We ensured that samples were randomly assigned to the training cohort and validation cohort during the grouping process. Moreover, there was no heterogeneity between the two cohorts in terms of mortality, tumor grade, clinicopathologic features, and age. A flowchart of this study is shown in Figure 1.

### lncRNA Annotations of CpG Sites

We annotated the genome of all CpG sites we obtained to characterize lncRNAs associated with the above methylation sites. The correlation between the lncRNA methylation level and specific methylation sites was investigated in the total cohort. Then, we analyzed the methylation level of lncRNA promoter region in all glioma samples and drew a heat map according to the methylation classification of lncRNAs in the glioma samples.

### Univariate and Multivariate Survival Analyses of lncRNA

Univariate Cox analysis was performed using the survival coxph function of the R package (http://rstudio.com) to explore the correlation between the methylation degree of lncRNA and prognosis, with *p* < 0.05 as the threshold. Subsequently, the age, sex, and WHO grade of glioma patients were analyzed for their correlation with prognosis. The methylated lncRNAs promoter regions that were substantially related to prognosis were selected for multivariate regression analysis, with *p* < 0.05 as the significance threshold.

### Construction of Risk Prognosis Signature

In the multivariate Cox regression analysis, the regression coefficients of the nine prognostic lncRNAs were obtained by using the R-package “Survival.” Next, we incorporated nine screened lncRNAs into the following formula to determine each patient's risk score. The risk score = αlncRNA1 × βlncRNA1 + αlncRNA2 × βlncRNA2 + … + αlncRNA9 × βlncRNA9. The α-values are the regression coefficient of lncRNA, which represents the contribution of lncRNA to the prognostic risk score. The β-values were used to represent the methylation level of lncRNA promoter region. Then, we constructed a prognostic risk signature for glioma patients. According to the risk score of each glioma patient, the corresponding median risk score was used as the critical value to divide glioma patients into the low-risk and high-risk groups. We used the Kaplan-Meier curve to assess survival differences in glioma patients between the high-risk and low-risk groups.

### Functional Enrichment Analysis

Gene ontology (GO) and Kyoto Encyclopedia of Genes (KEGG) analyses were performed to predict the function of these differentially methylated sites. Gene ontology and Kyoto Encyclopedia of Genes analyses used the R programming language to study the cell functions associated with the risk factors defined by the signature of nine lncRNA methylated genes in the TCGA database.

### Cells Culture

All glioma cell lines were purchased from the Cell Bank of the Chinese Academy of Sciences (Shanghai, China). The cell lines were as follows: one normal HEB cell line and six glioma cell lines (LN18, SF126, T98G, SNB19, SW1088, and U251). The cells were cultured in DMEM (Gibco, USA) supplemented with 10% fetal bovine serum (Gibco, USA) in a constant temperature incubator with saturated humidity and 5% CO_2_ at 37°C, and the cells grew as a monolayer. The average time of fluid exchange was 48–72 h. Observed under the inverted phase contrast microscope, when the adhesion reached more than 80%, the cells were digested and passaged with trypsin.

### Silencing of lncRNA and Transfection

We used the lncRNA Smart Silencer and its negative control, lncRNA smart silencer NC synthesized by RiboBio (Guangzhou, China) to knockdown PCBP1-AS1 and LINC02875. The Smart Silencer was a mixture of three small interference RNAs (siRNAs) and three antisense oligonucleotides (ASOs), which could effectively knock down both nuclear and cytoplasmic lncRNAs. Cells in the logarithmic growth phase were selected, inoculated evenly in a 6-well plate, and prepared for transfection when the cell growth confluence reached 30–50%. Transfections were conducted and finished by using riboFECT™ CP Reagent (Guangzhou, China). Diluted 10 μl siRNA/NC-RNA in 120 μl 1X riboFECT™ CP Buffer and 12 μl riboFECT™ CP Reagent, mixed fully and incubated for 15 min at room temperature. Add the mixture of riboFECT™ CP to the 6-well plate and mix gently. After 24 h, the medium was replaced with DMEM containing 10% serum to continue the culture.

### qRT-PCR

The following RT-qPCR primers were applied:

GAPDH, forward 5′-AGCAAGAGCACAAGAGGAAG-3′, reverse 5′-GGTTGAGCACAGGGTACTTT-3′.
PCBP1-AS1, forward 5′-TTCTCGGTGACTCCATTTCC CA-3′, reverse 5′-TGGGTCAACACCTCTCATCA-3′.
LINC02875, forward 5′-CTTGAGGGACAGCCTCTACG-3′, reverse 5′-GGACTGGATTTTGGGTCCTT-3′.

TRIzol reagent (Takara, Japan) was used to extract total cellular RNA from transfected LN18 and T98G cells. According to the reverse transcription kit PrimeScript RT Master Mix (Takara, Japan) instructions, cellular RNA was reverse transcribed into cDNA. Next, TB Green Premix Ex Taq II (Takara, Japan) was utilized for real-time PCR. cDNA was used as the template for PCR, with 1.6 μl substrate, 3.2 μl primer for PCR, and 5.2 μl TB Green. The steps for RT-qPCR reaction were as follows: pre-denaturation at 95°C, 30 s, one cycle; quantitative analysis at 95°C, 5 s, 60°C, 31 s, 40 cycles; dissolution curve at 95°C, 15 s, 60°C, 1 min, 95°C, 15 s, one cycle. The expression level of the target gene was normalized to that of the control GAPDH and calculated using the following formula: 2-ΔΔCt.

### Cell Viability Assay

Cell viability was calculated by using an MTT kit (Biyuntian, Shanghai). LN18 and T98G cells that had been transfected with si-PCBP1-AS1 and si-LINC02875 were grown in 96-well plates at a density of 6,000 cells per well. After incubation for 24 h, according to the instructions, cell viability was assessed by adding 10 μl of MTT dye (5 μg/μl) to each well. Next, the plates were kept in the dark for 4 h. Then, the media was removed carefully, and DMSO was added to each well. Finally, the plate was read using a microplate reader.

### Cell Migration and Invasion Assay

At 24 h post-transfection, LN18 and T98G cells were collected in a 6-well plate. A cell counting plate was used to count cells under an inverted phase contrast-microscope, and 0% DMEM was used to hydrate a Transwell chamber, where 2 × 10^4^ cells were inoculated. Six hundred microliters of 30% DMEM were added to the 24-well plate to cover the bottom of the chamber. After being cultured in a constant temperature cell incubator for 24 h, the chamber was soaked in 4% paraformaldehyde to fix the cells for 30 min and then stained with 0.1% crystal violet for 15 min. Cell invasion was assessed by adding Matrigel® (Gibco, USA) to the upper chamber, inoculating 10 × 10^4^ cells in the upper chamber, and then incubating for 48 h under the same culture conditions. Then, the Matrigel® was carefully swabbed onto the Matrigel® chamber. Finally, the number of cells passing through the small ventricular membrane was calculated under a phase-contrast inverted microscope (Olympus, Japan).

### Colony Formation Assay

A colony formation assay was used to further determine the proliferation ability of cells. The indicated LN18 and T98G cells and negative control cells were collected and counted with cell counting plates under an inverted phase-contrast microscope. Eight hundred cells were added to each of the 6-well plates. After a week of culture, the cells were stained with 0.1% crystal violet (Biyuntian, Shanghai), fixed with 4% paraformaldehyde (Biyuntian, Shanghai) for 30 min, and photographed under white light to calculate the number of cell clones in each well.

### Statistical Analysis

All experimental data are expressed as the mean ±*SD* conducted at least three times, and GraphPad Prism 8 (La Jolla, USA) was used for statistical analysis of experimental data. Statistical significance between different groups was assessed using ANOVA or Student's *t*-test (two tails). *P*-values were calculated using Student's *t*-test comparison group or log-rank test for Kaplan-Meier curves. *P*-values of < 0.05 were considered statistically significant.

## Results

### Patient Characteristics

We downloaded the methylation 450K data and clinical-pathological data of glioma patients from the TCGA database. All clinical follow-up data and methylation data obtained were investigated for eligibility, and a total of 675 glioma patients had corresponding prognostic information. After our comparison, we found that 649 samples were successfully matched. A series of pretreatments were performed on the obtained methylation data to explore the candidate CpG sites of promoter regions in the total glioma samples. These measures include finding differentially methylated sites and differentially methylated regions, completing missing values, removing samples with incomplete data, and excluding CpG sites with genomic cross-reactivity. Then, we obtained the unique CpG sites and annotated each CpG site with lncRNA. Finally, a total of 286 lncRNAs were identified.

### Analysis of the Methylation Level of lncRNAs Promoter Region in Different Grades of Glioma Samples and Classification Based on Prognosis

**Figure 2:**
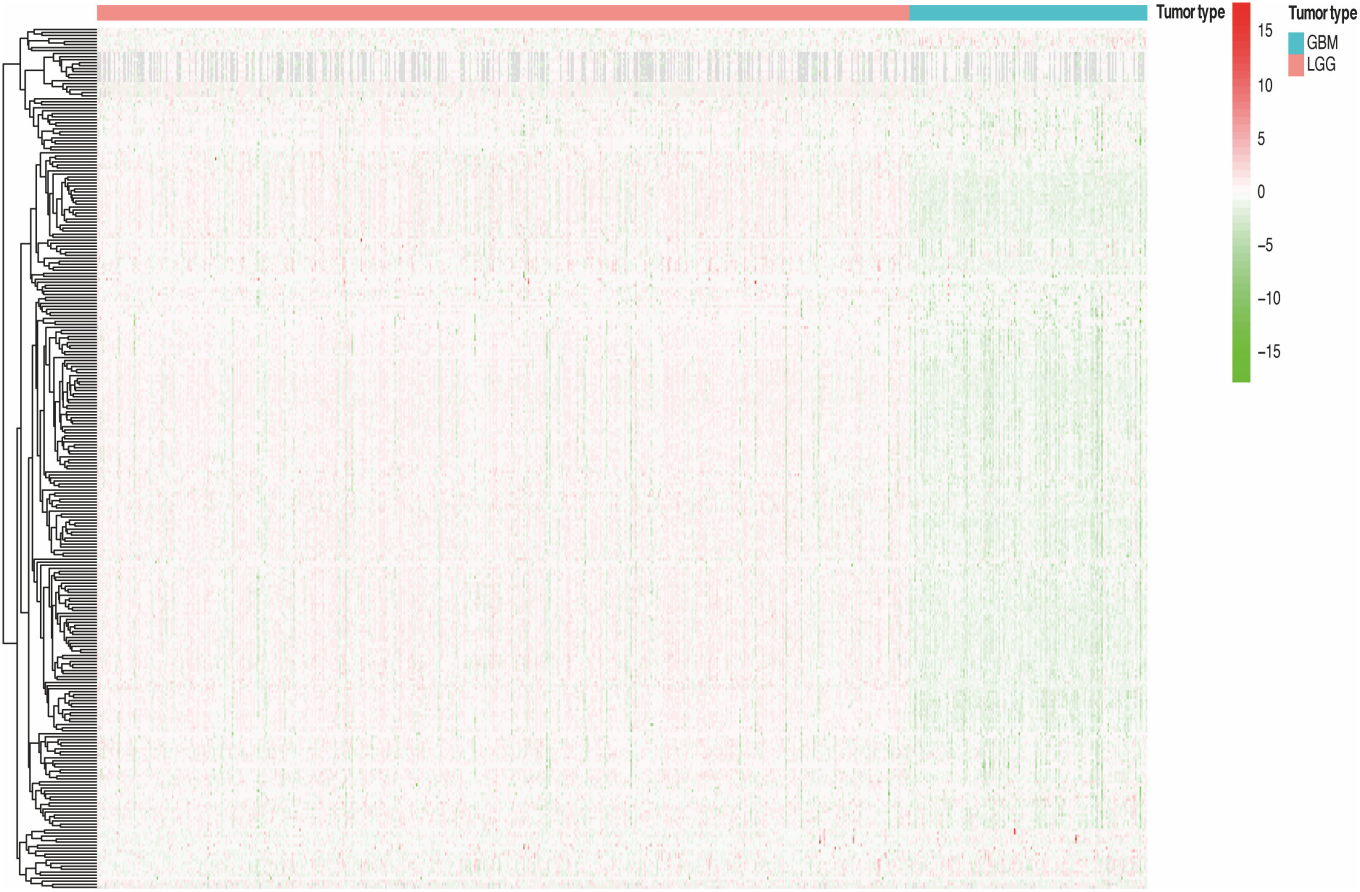
The methylation degree of lncRNA promoter regions of in GBM and LGG samples.

We divided all glioma samples into low-grade glioma groups and high-grade glioma groups based on their clinical characteristics. In the total cohort, the WHO grade of the glioma samples and the methylation level of lncRNA promoter region are presented in the heat map (Figure 2). We found that the methylation level of lncRNA promoter region was significantly correlated with the WHO grade of glioma samples. Furthermore, the methylation level of lncRNA promoter region in the high-grade glioma samples was significantly higher than that in the low-grade glioma samples. Considering the potential role of a single methylated lncRNA promoter region in glioma patients, we systematically investigated the relationships between the methylation status of each lncRNA promoter region and the pathological features of glioma, including WHO grades, ages, genders, and survival. Next, we performed a univariate Cox proportional hazard regression analysis in the total set of glioma samples. We found that WHO grade, tumor type, and age were closely related to the prognosis of the patients. Then, we randomly assigned all samples to the training or validation cohort. Next, we investigated the relationship between the methylation level of lncRNA promoter region and glioma patient survival in the training cohort (*n* = 325) and verified the results in the validation cohort (*n* = 324).

### Prognostic Value of the lncRNA Promoter Region Methylation and a Risk Signature Built Using Nine Selected lncRNAs

**Figure 3:**
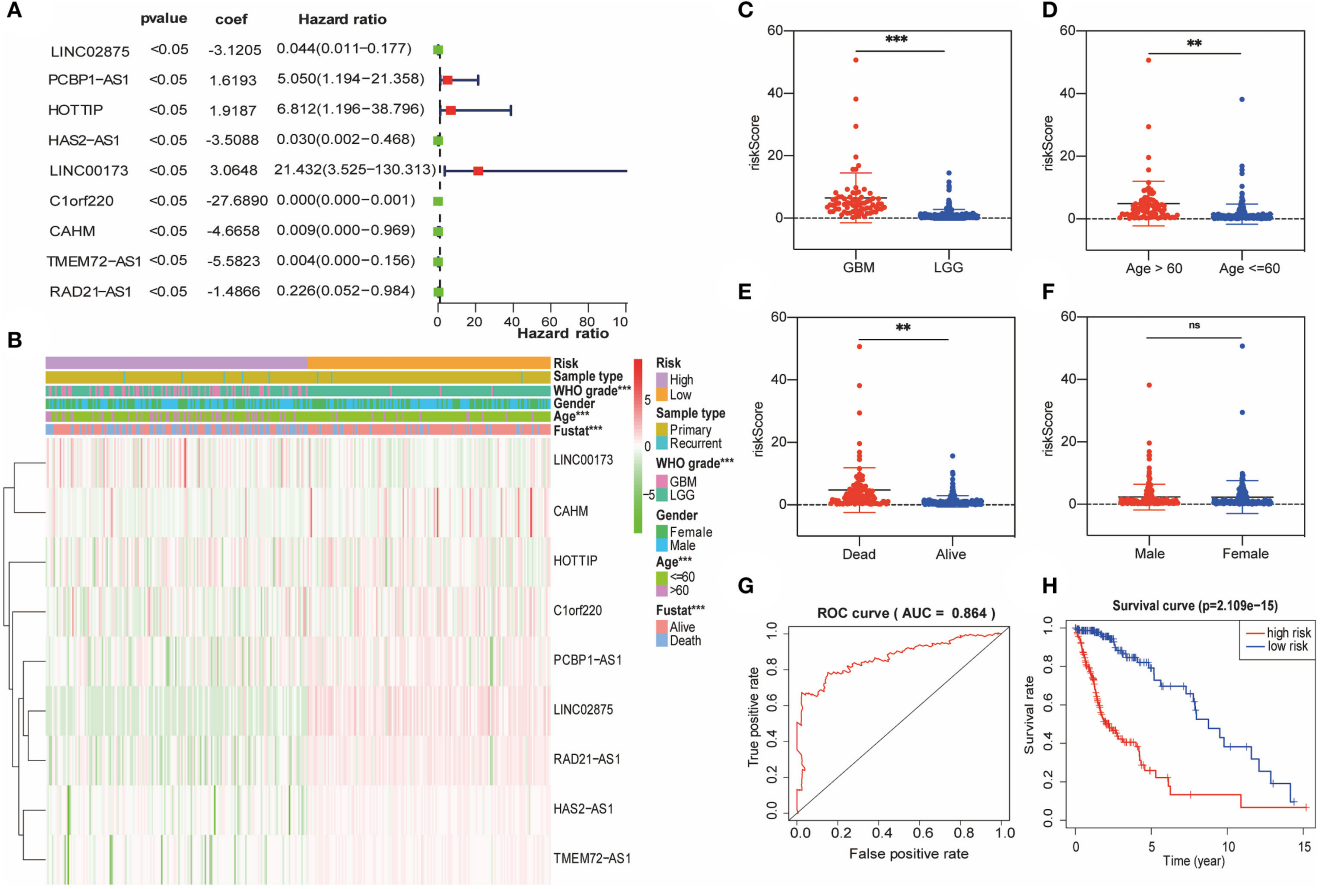
A risk signature with the nine lncRNA methylated genes. **(A)** The forest plot represents the multivariate Cox analysis of nine lncRNAs with prognostic characteristics. The coef value represents the regression coefficient. **(B)** The heat map shows the difference between the methylation classification and clinicopathological characteristics of nine lncRNAs in the low-risk and high-risk groups. **(C–F)** Boxplots show the risk assessment score of glioma patients with different WHO grades **(C)**, ages **(D)**, survival statuses **(E)**, and genders **(F)**. **(G)** ROC curve of the high-risk and low-risk groups validated by the nine lncRNA methylated genes signature. **(H)** Kaplan-Meier survival curves of the patients assigned to high-risk and low-risk groups based on the risk score. **p < 0.01, ***p < 0.001; ns, no significance.

Subsequently, we attempted to investigate the prognostic role of lncRNA promoter region methylation in glioma patients. In the training cohort, we performed a univariate Cox regression analysis of the methylation level of lncRNA promoter region in glioma samples. Finally, we found that nine lncRNA methylated genes (LINC00173, HOTTIP, CAHM, PCBP1-AS1, HAS2-AS1, TMEM72-AS1, RAD21-AS1, C1orf220, and LINC02875) were associated with the prognosis of the patient (Figure 3A). The regression coefficients of the nine best prognostic lncRNAs were obtained through the multivariate Cox proportional hazard regression model using the R-package “Survival,” and the promoter methylation levels and coefficients of each lncRNA were combined by linear combination. Finally, we established the following risk prediction model: risk score = (3.0648 × promoter methylation level of LINC00173) + (1.9187 × promoter methylation level of HOTTIP) + (−4.6658 × promoter methylation level of CAHM) + (1.6193 × promoter methylation level of PCBP1-AS1) + (−3.5088 × promoter methylation level of HAS2-AS1) + (−5.5823 × promoter methylation level of TMEM72-AS1) + (−1.4866 × promoter methylation level of RAD21-AS1) + (−27.6890 × promoter methylation level of C1orf220) + (−3.1205 × promoter methylation level of LINC02875). Then, we separated the glioma samples into low-risk and high-risk groups based on the median risk score in the training cohort (Figure 3B). The association between the risk score and clinicopathological characteristics of each glioma sample was investigated. We discovered considerable differences between the high-risk and low-risk groups in WHO grade (Figure 3C), patient age (Figure 3D), and survival (Figure 3E), but not by sex (Figure 3F) and IDH1 mutation status (Supplementary Figure 1A). Compared with the low-risk group, the number of glioma patients in the high-risk group, their age, and the WHO grade of glioma were relatively higher. The area under the receiver operating characteristic (ROC) curve was used to assess the prognostic accuracy of the nine lncRNA methylated genes signature. The results showed that the AUC of the signature was 0.864 (Figure 3G), suggesting that the risk prediction model we constructed had high specificity and sensitivity. In addition, we also observed significant differences in prognosis between the two categories. In the training cohort, the glioma patients in the high-risk score group had worse survival than those in the low-risk score group (Figure 3H).

### The Risk Score Was Strongly Related to the Clinicopathological Characteristics of Glioma Patients

**Figure 4:**
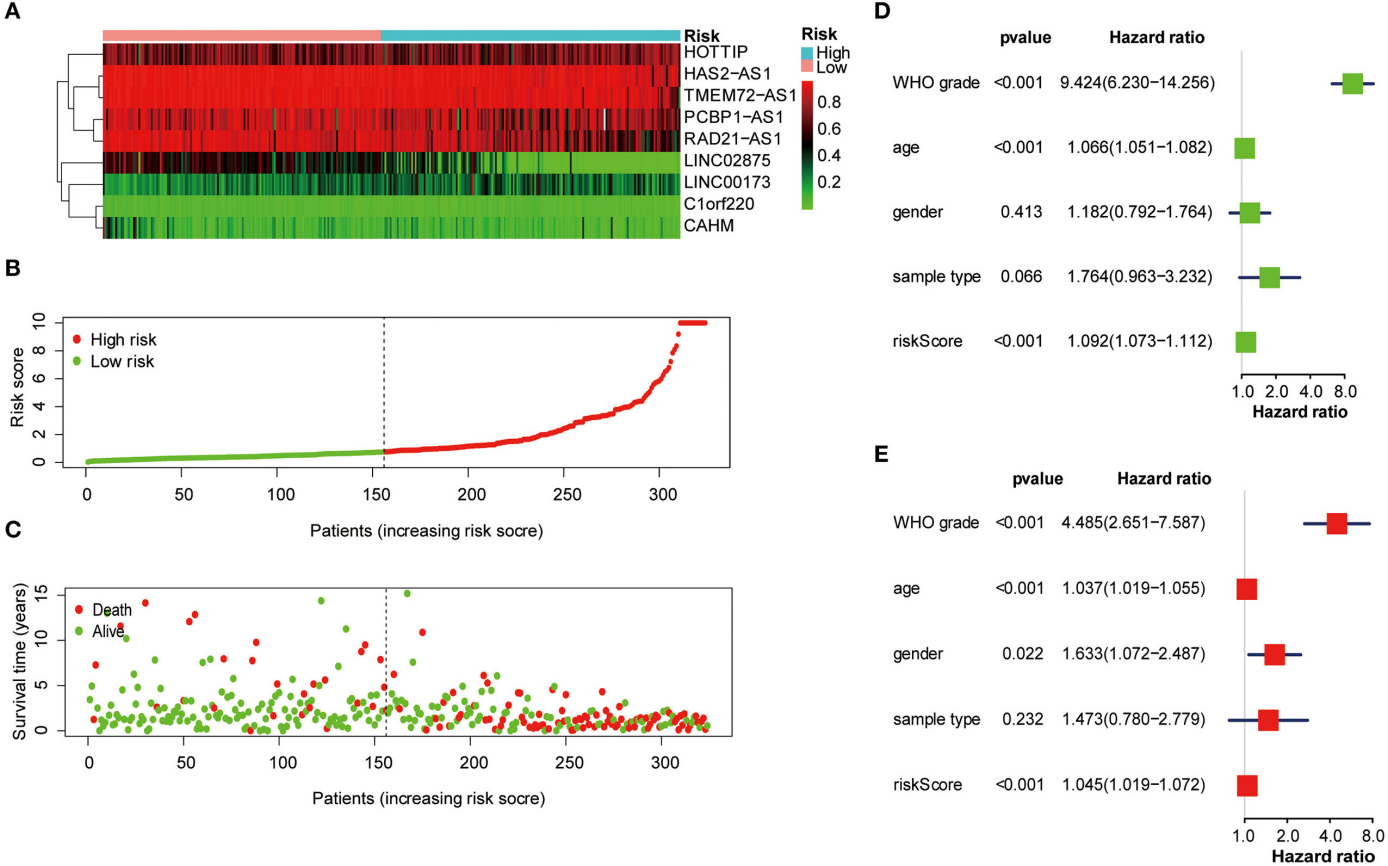
The nine lncRNA methylated genes signature was used to analyze the survival time and survival status of glioma patients. **(A)** Heat map of the methylation classification of the nine promoter regions methylated lncRNAs in glioma patients. **(B)** Distribution of risk scores for each glioma patient. The median risk score was used as critical point. The red circles represent the high-risk group, and the green circles represent the low-risk group. **(C)** The scatter diagram shows the survival of glioma patients. The red circles represent deceased individuals; the green circles represent living individuals. **(D, E)** Univariate **(D)** and multivariate **(E)** Cox regression analyses of the correlation between the risk score and clinicopathological factors.

Next, the nine lncRNA methylated genes were used to analyze the survival time and survival status of glioma patients. The promoter region methylation degree of the nine selected lncRNAs, risk score distribution, and survival status are shown in Figures 4A–C. Univariate and multivariable Cox regression analyses were performed in the training cohort to determine whether the nine lncRNA methylated genes signature is an independent indicator predictor that could predict the prognosis of glioma patients. Univariate analysis showed that the risk score, age, and WHO grade of glioma patients significantly influenced their prognosis (Figure 4D). Then, we incorporated these factors into multivariate Cox regression, and the results showed that only risk score and WHO grade were significant for the prognosis of glioma patients (Figure 4E). Therefore, these results confirmed that the risk score derived from nine lncRNA methylated genes could independently predict prognosis in glioma patients.

### Verification of the Nine lncRNA Methylated Genes Signature in the Validation Cohort

**Figure 5:**
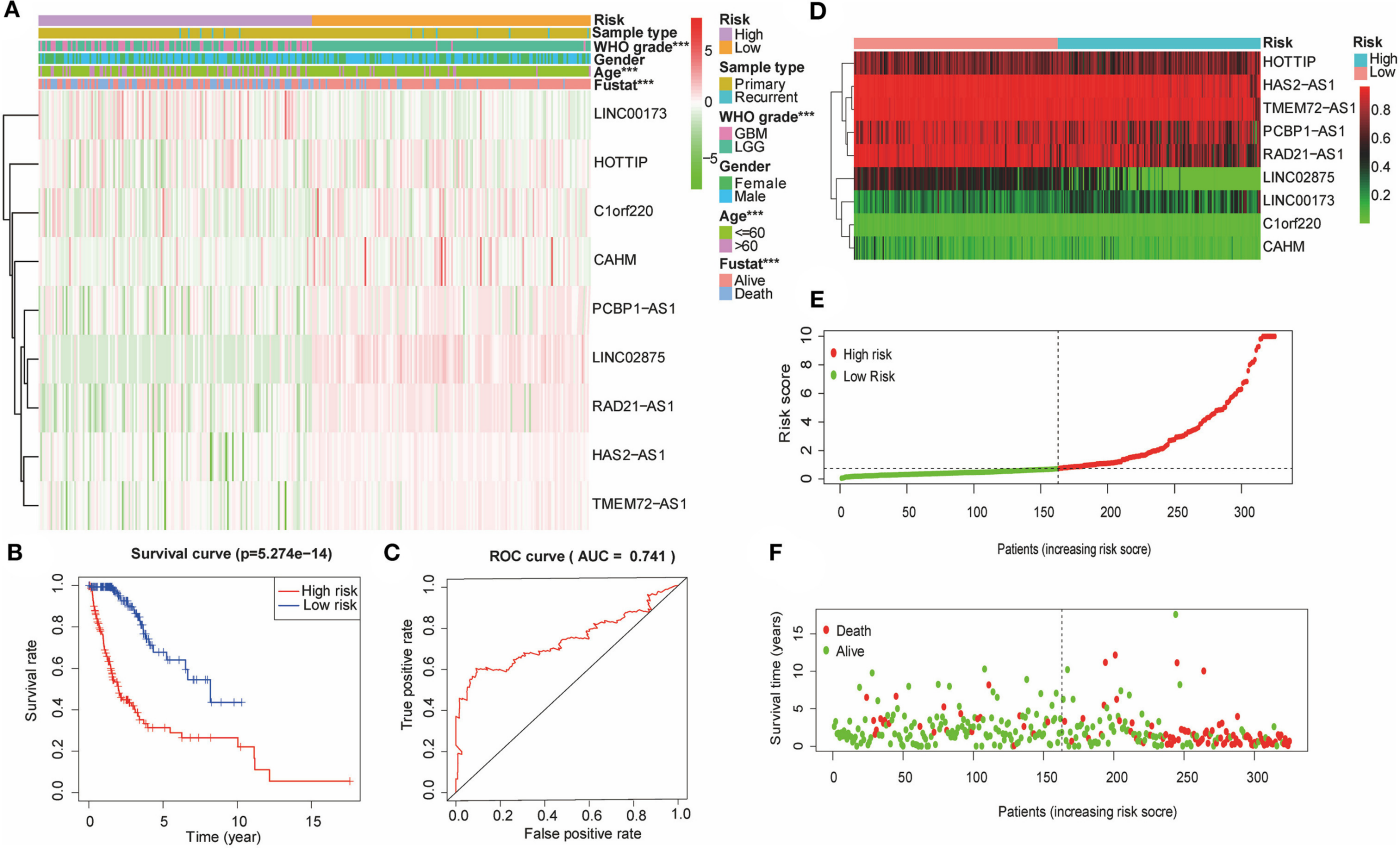
The nine lncRNA methylated genes signature divided glioma patients into high-risk and low-risk groups with different prognoses in the validation cohort. **(A)** The heat map shows the difference between the methylation classification and clinicopathological characteristics of the nine lncRNA methylated genes in the low-risk and high-risk groups. **(B)** Kaplan-Meier survival curves of the patients assigned to high-risk and low-risk groups based on the risk score. **(C)** ROC curve of the high-risk and low-risk groups validated by the nine lncRNA methylated genes signature. **(D)** Heat map of the promoter region methylation classification of the nine lncRNAs in the validation cohort. **(E)** Distribution of risk scores for each glioma patient. The median risk score was used as critical point. The red circles represent the high-risk group, and the green circles represent the low-risk group. **(F)** The scatter diagram shows the survival of glioma patients. The red circles represent deceased individuals; the green circles represent living individuals. ***p < 0.001.

Based on the previously constructed signature, multiple Cox regression analysis was used to score the risk of the validation cohort (*n* = 324). According to the median risk score, the glioma samples in the validation cohort were divided into high-risk and low-risk groups (Figure 5A). The R software survival package was used to draw survival curves to compare the survival time of the two groups. The results showed that the WHO grade and the age of glioma patients in the high-risk group were higher than those in the low-risk group. Furthermore, the high-risk group had a worse prognosis than the low-risk group (Figure 5B). The ROC curve was drawn to verify the reliability of the signature, and the area under the curve (AUC) value was 0.763 (Figure 5C). Then, we plotted a scatter plot based on the risk score of patients, showing survival in glioma patients with different risk scores (Figures 5D–F). The results showed that the number of survivors decreased with the increase in glioma patients' risk score, which was consistent with what we observed in the training cohort. Therefore, these results suggested that the nine lncRNA methylated genes signature is an effective predictor of the prognosis of glioma patients in the validation cohort.

### Potential Biological Processes Associated With the High-Risk Group as Classified by the Signature

**Figure 6:**
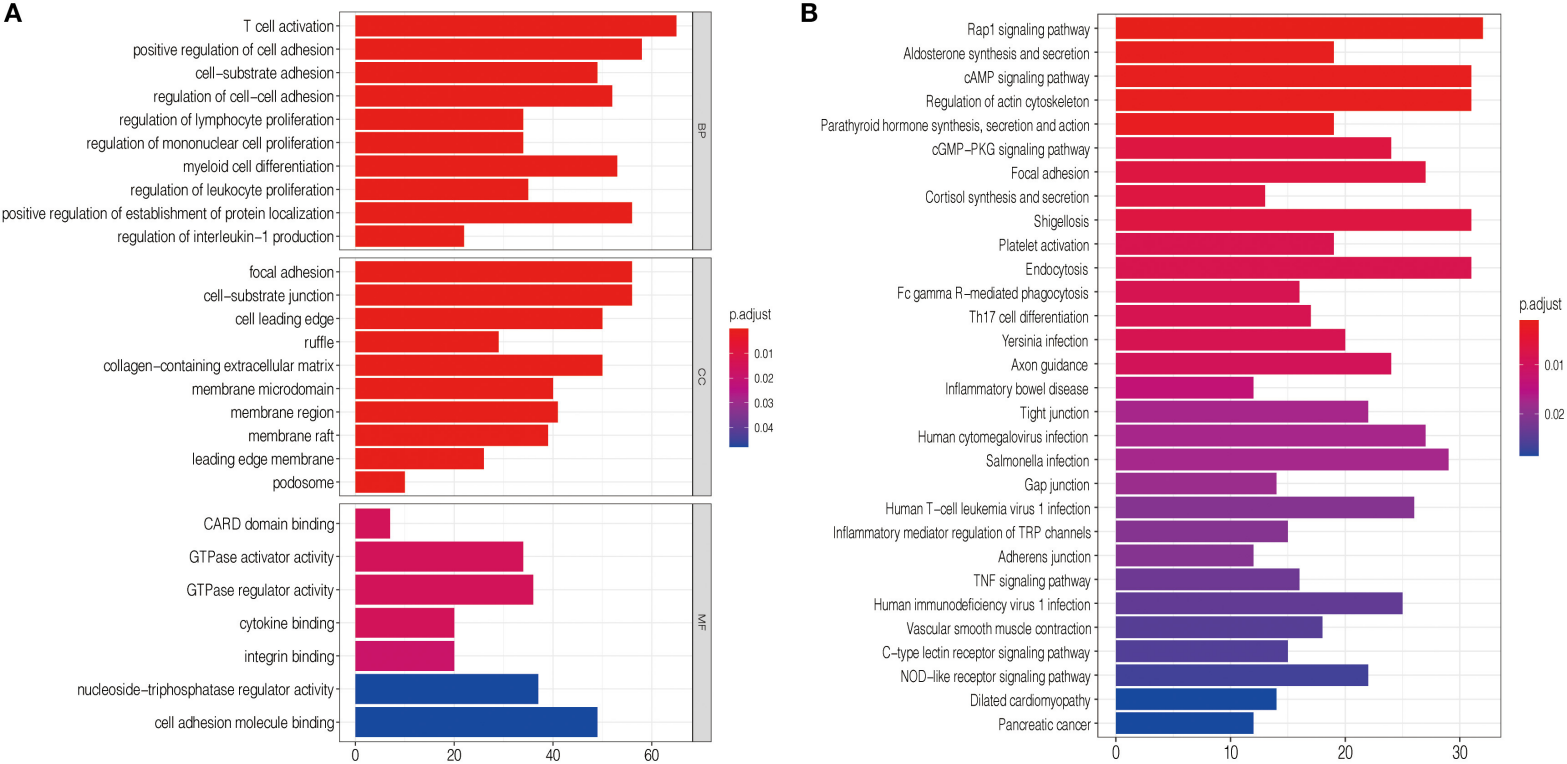
Functional analysis of the nine lncRNA methylated genes signature. **(A)** GO annotations based on the top 4,400 genes positively associated with the nine lncRNA methylated genes signature. **(B)** KEGG pathways positively correlated with the risk score of glioma patients.

To explore the functional characteristics of potential changes associated with the nine lncRNA methylated genes signature, GO analysis was performed to investigate the correlation of the different functions between the high-risk and low-risk groups. In the high-risk group, we discovered that the positively related genes were mainly enriched in cell-substrate adhesion, positive regulation of cell adhesion, and focal adhesion, closely associated with malignant phenotypes such as migration and invasion of glioma (Figure 6A). Next, KEGG pathway enrichment analysis was used to verify that the high-risk group was strongly related to malignant glioma phenotypes. We observed that the Rap1 signaling pathway, cAMP signaling pathway, cGMP–PKG signaling pathway, and TNF signaling pathway were closely associated with the high-risk group defined by the signature (Figure 6B). The mechanism of these cell signaling pathways in promoting the malignant phenotype of glioma has been studied previously (29–32).

### The lncRNA Methylated Genes Signature Showed Significant Differences in Expression in Normal Brain Tissue and Glioma Tissue and Had Different Prognoses in Glioma Patients

**Figure 7:**
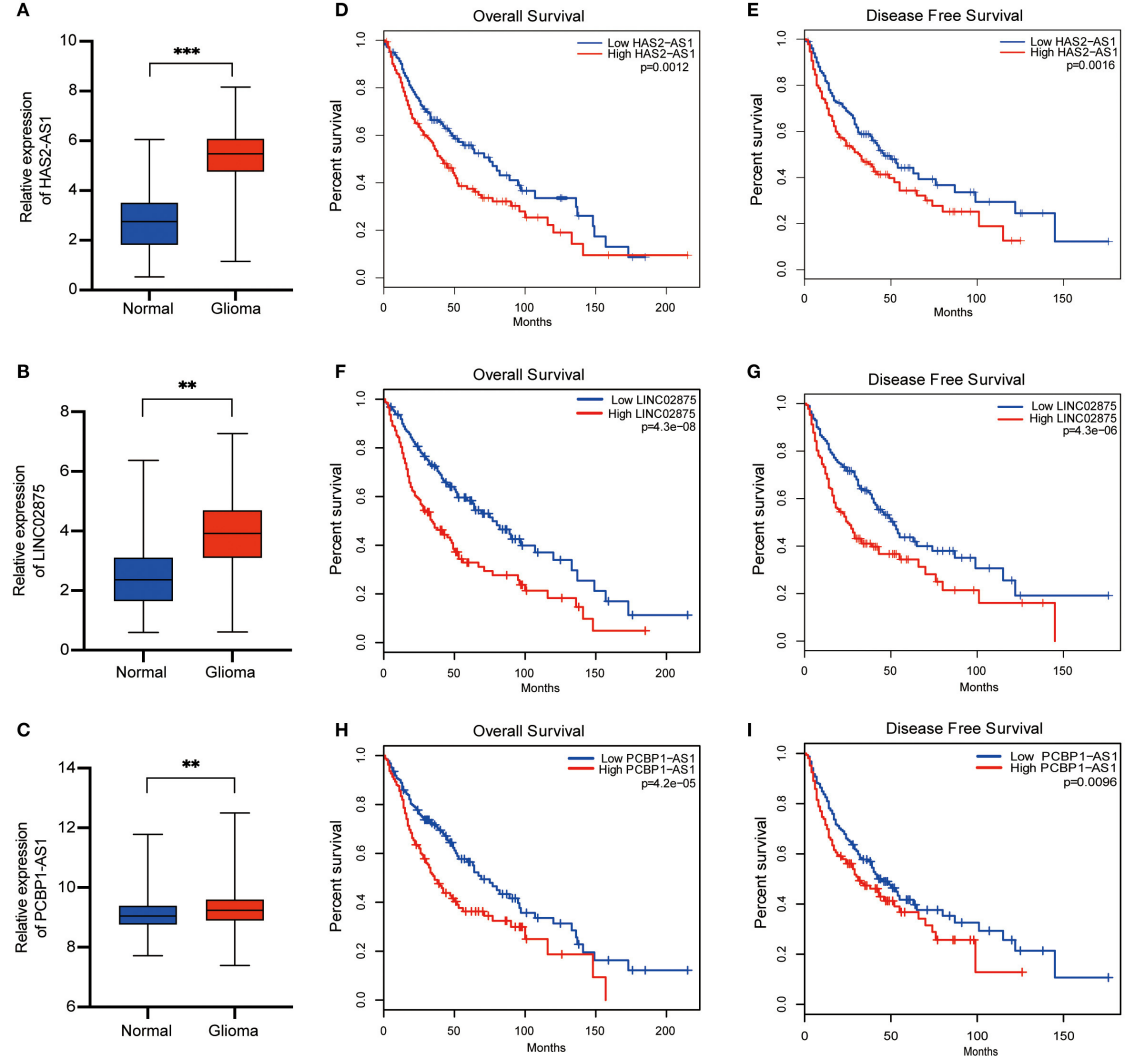
Differential expression and prognosis of the three selected lncRNAs. **(A-C)** Boxplots show the relative expression levels of HAS2-AS1 **(A)**, LINC02875 **(B)**, and PCBP1-AS1 **(C)** in normal brain tissue and glioma. The Kaplan-Meier curve for OS **(D)** and DFS **(E)** of glioma patients with high HAS2-AS1 expression and low HAS2-AS1 expression using TCGA data. The Kaplan-Meier curve for OS **(F)** and DFS **(G)** of glioma patients with high LINC02875 expression and low LINC02875 expression using TCGA data. The Kaplan-Meier curve for OS **(H)** and DFS **(I)** of glioma patients with high PCBP1-AS1 expression and low PCBP1-AS1 expression using TCGA data. **p < 0.01; ***p < 0.001.

To further verify the accuracy of the risk signature we constructed, nine lncRNA methylated genes were analyzed in glioma and normal brain tissue from TCGA and GTEX, respectively. We found that five out of the nine selected lncRNAs were upregulated in glioma tissue, including CAHM, PCBP1-AS1, HAS2-AS1, RAD21-AS1, and LINC02875 (Figures 7A–C). Then, the Gene Expression Profiling Interactive Analysis (GEPIA, http://gepia.cancer-pku.cn/) database was analyzed to generate survival curves and hazard ratios for these nine selected lncRNAs (33). We found that the differential expression of nine lncRNAs had different overall survival (OS) and disease-free survival (DFS) rates in glioma patients. In addition, the hazard ratios of three lncRNAs (HAS2-AS1, PCBP1-AS1, and LINC02875) were >1 and correlated with the prognosis of glioma patients (Figures 7D–I and Supplementary Figure 2). Therefore, these results suggested that HAS2-AS1, PCBP1-AS1, and LINC02875 may play an important role in the malignant progression of glioma.

### Knocking Down of PCBP1-AS1 and LINC02875 Can Significantly Inhibit the Proliferation, Migration, and Invasion of Glioma Cells

**Figure 8:**
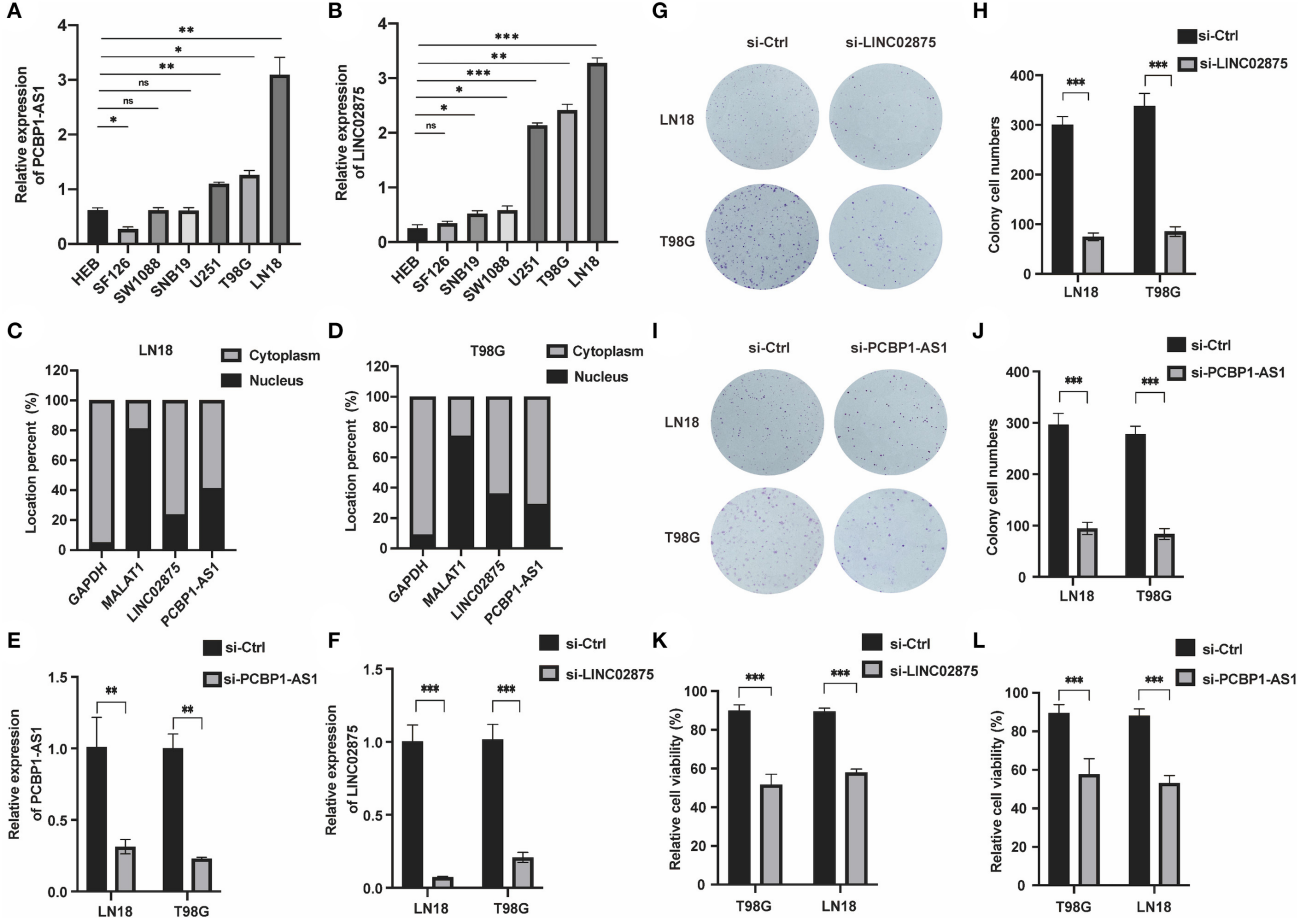
Knockdown of PCBP1-AS1 and LINC02875 inhibited the activity and proliferation of glioma cells. Relative expression of PCBP1-AS1 **(A)** and LINC02875 **(B)** in seven cell lines. The expression location of PCBP1-AS1 and LINC02875 in LN18 **(C)** and T98G **(D)** cells. qRT-PCR to detect the relative silencing levels of PCBP1-AS1 **(E)** and LINC02875 **(F)** in LN18 and T98G cells. Representative imaging **(G,I)** or counting **(H,J)** of the colonies formed by LN18 and T98G cells after silencing with PCBP1-AS1 and LINC02875 for 10 days. Cell proliferation was measured by the MTT assay after silencing with LINC02875 **(K)** and PCBP1-AS1 **(L)** in LN18 and T98G cells. *p < 0.05; **p < 0.01; ***p < 0.001; ns, no significance.

**Figure 9:**
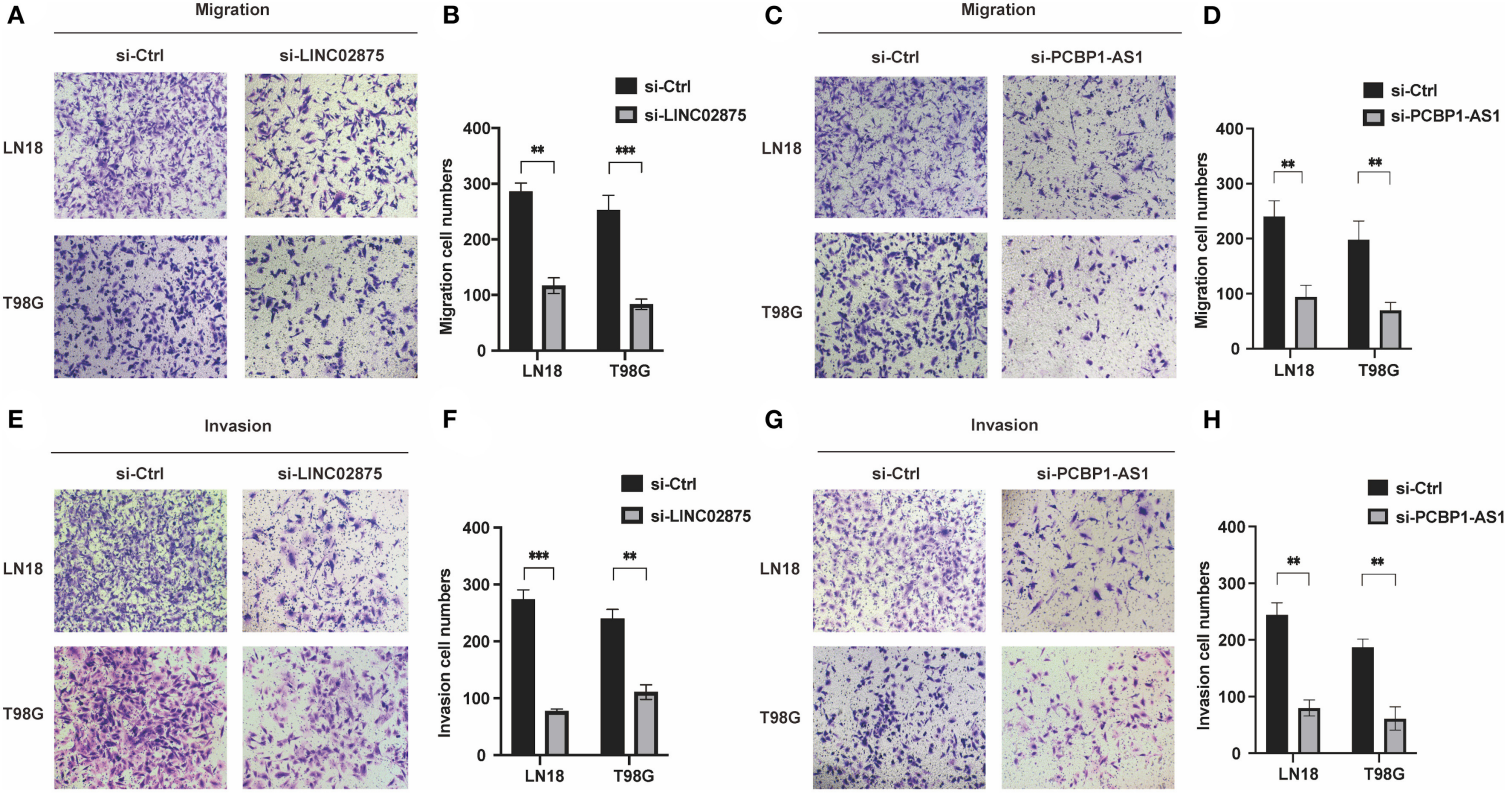
Downregulation of PCBP1-AS1 and LINC02875 decreased the migration and invasion of glioma cells. Representative imaging **(A,C,E,G)** or counting **(B,D,F,H)** of migration assays after silencing PCBP1-AS1 and LINC02875 in glioma cells. Scale bar, 200μm. **p < 0.01; ***p < 0.001.

We further analyzed the three lncRNAs and found that only HAS2-AS1 has been reported in gliomas (34), while the functions of PCBP1-AS1 and LINC02875 have not been researched in gliomas. Therefore, we chose PCBP1-AS1 and LINC02875 as the research subjects to illustrate their functions in gliomas. The RT-PCR assay was used to analyze the expression levels of PCBP1-AS1 and LINC02875 in different glioma cell lines, and the results showed that the PCBP1-AS1 and LINC02875 expression levels were relatively high in LN18 and T98G glioma cells (Figures 8A,B). Next, we analyzed the locations of PCBP1-AS1 and LINC02875 in glioma cells. Nucleocytoplasmic fractionation was performed, and the lncRNA levels in nuclear and cytoplasmic fractions were quantified by RT-PCR. The results showed that PCBP1-AS1 and LINC02875 were both expressed in the nucleus and cytoplasm (Figures 8C,D). Therefore, we used Smart Silencer to silence the expression of PCBP1-AS1 and LINC02875 in glioma cells (Figures 8E,F). The RT-PCR demonstrated that the expression levels of PCBP1-AS1 and LINC02875 were significantly downregulated in LN18 and T98G cells. We first conducted a clone formation experiment, and the results showed that after knocking down PCBP1-AS1 and LINC02875, the clone formation ability of LN18 and T98G cells was significantly decreased (Figures 8G–J). Next, we analyzed cell viability by assessing the MTT assay and found that the viability of LN18 and T98G cells decreased after silencing PCBP1-AS1 and LINC02875 compared with that of the negative control cells (Figures 8K,L). Finally, we performed cell Transwell assays and Matrigel invasion assays. The results showed that knocking down PCBP1-AS1 or LINC02875 expression led to a substantial decrease in the migration and invasion ability of glioma cells (Figure 9). In summary, our results indicated that knocking down the expression of PCBP1-AS1 or LINC02875 could reduce glioma cell proliferation, migration, and invasion. In addition, we tried to explore the relationship between the methylation level of the lncRNA promoter region and IDH1 mutations in glioma samples. However, our results showed that here was no statistical difference in the promoter methylation levels of LINC02875 and PCBP1-AS1 in glioma samples with different IDH1 mutation states (Supplementary Figures 1B–J).

## Discussion

High malignancy and recurrence rates make glioma the most lethal primary brain tumor of the central nervous system (35, 36). With the development of glioma mechanism research and clinical treatment, some prognostic factors have been well-characterized, including WHO grade, isocitrate dehydrogenase (IDH) status, and 1p/19q codeletion status. Epigenetics mainly involves the modification of DNA or proteins and post-translational modification of histone proteins, and its function in the occurrence, malignant progression, and prognosis of gliomas has been demonstrated (37, 38).

DNA methylation is an important epigenetic modification that maintains genome integrity and regulates gene expression (39). The genome-wide DNA methylation environment of glioma has shown extensive spatiotemporal heterogeneity. Through whole-genome DNA methylation analysis, researchers can distinguish the malignant degree of gliomas and predict the prognosis of patients (40, 41). LncRNA, as a new participant, presented a new approach to explore the biological function of glioma cells and predict the prognosis of glioma patients (42). Moreover, methylated lncRNAs can directly bind to transcription factors or act as ceRNAs and bind to miRNAs, which plays an epigenetic modification role in tumors (24, 43). Nevertheless, the abnormal changes in DNA methylation patterns of lncRNAs have not been further studied. Similar to genes with coding functions, lncRNA expression is also affected by copy number changes and epigenetic modifications. In addition, promoter regulation and gene quantification can also affect the overall expression level of lncRNAs (44, 45). Epigenetic regulation of lncRNAs has been suggested to be an important mechanism contributing to tumor progression (46). Among them, lncRNA expression could be influenced by changes in the methylation level of its promoter or enhancer region, thus promoting the malignant progression of glioma. Liu et al. indicated that hypermethylation of the LINC00261 promoter region leads to its low expression in pancreatic cancer and is associated with a worse prognosis (24). In esophageal squamous cell carcinoma, hypermethylation of the MEG3 promoter has been observed, which is related to the downregulation of MEG3, and after treatment with the DNA methyltransferase inhibitor 5-Aza-dC, this process can be reversed (43). As described above, the expression level of lncRNA in tumors is related to the level of methylation of its promoter region.

High-throughput biological techniques based on human genome detection have been widely used to predict the malignancy and prognosis of glioma (47, 48). Recently, a research explored specific prognosis-subtypes based on DNA methylation status using GBM samples from TCGA database and constructed a risk model that could predict the prognosis of GBM patients using 10 CpG sites (49). LncRNA, as novel biological prognostic markers, has also attracted the attention of researchers. Deng et al. identified an optimal methylated lncRNA marker for prognosis in osteosarcoma patient and established a methylated lncRNA signature that is significantly associated with survival in osteosarcoma patient (26). However, to date, the use of lncRNA promoter regions methylation to construct a risk signature has not been reported in glioma.

Therefore, it is indispensable to establish a lncRNA methylated genes signature to predict the prognosis of patients with different grades of glioma. In this study, we constructed a prognostic risk signature with nine lncRNA methylated genes. The signature successfully divided glioma samples into high-risk and low-risk groups. We observed notable differences between the high-risk and low-risk groups with respect to WHO grade, age, and survival. Due to the possibility of overtraining or false positives in the nine lncRNA methylated genes. Then, we constructed a validation cohort and further verified the prognostic value of the prognostic signature in the validation cohort. Similar to the result of the training cohort, the result of the validation set showed that the signature has excellent accuracy and repeatability.

Our study found that most of the selected lncRNAs in the samples were in a state of hypomethylation of promoter region in the high-risk group. Moreover, in the high-risk group, the number of glioma patients was relatively higher, and the patients' prognosis was also poor. These results indicated that the lncRNA methylated genes we selected are basically tumor-promoting, and hypomethylation of the promoter region leads to upregulation of their expression, which leads to the occurrence of high-grade gliomas. Lu et al. demonstrated that loss of DNA methylation makes the promoter of lncRNA more accessible to this transcription factor, which leads to the upregulation of lncRNA expression and affects the survival status of glioma patients (27). Moreover, GO analysis and KEGG analysis also confirmed that the nine lncRNA methylated genes signature was strongly associated with tumor cell adhesion and signaling pathways related to the malignant progression of gliomas in the high-risk group. Based on the analysis, we selected two lncRNAs to verify our results through functional experiments in glioma cells. Loss-of-function assays confirmed that knockdown of PCBP1-AS1 and LINC02875 inhibited the proliferation, migration, and invasion of glioma cells. Therefore, this study explored the signature for the OS of glioma patients and provided a novel potential biomarker or therapeutic target for glioma.

However, the factors that mediate methylation of the lncRNA promoter region have not been thoroughly studied. Our next research direction will be to explore these factors. Although our study identified the innovation of the nine lncRNA methylated genes signature and verified the accuracy of the model in the validation set, the study still has limitations. First, our data lacked some clinicopathologic features associated with glioma prognosis, such as the location of the glioma and its molecular subtypes (classical, mesenchymal, neural, and proneural). Next, other common glioma biomarkers, such as MGMT status, 1q/19q codeletion and ATRX loss, were not included in the analysis of the signature. Nevertheless, compared with previous studies on lncRNA promoter region methylation, our study emphasized the critical roles of lncRNA promoter region methylation in glioma and provided valuable candidates for prognostic biomarkers and potential targets for glioma patients.

## Data Availability Statement

The original contributions presented in the study are included in the article/Supplementary Material, further inquiries can be directed to the corresponding author/s.

## Author Contributions

EB and BZ designed this article. MC and LS were responsible for data analysis and conducting practical experience. KH was responsible for recording the experience results. XY was in charge of article figure. JC and ZZ drafted the article. All authors contributed to the article and approved the submitted version.

## Conflict of Interest

The authors declare that the research was conducted in the absence of any commercial or financial relationships that could be construed as a potential conflict of interest.

## Supplementary Material

Supplementary Figure 1 **(A)** Boxplots show the risk assessment score of glioma patients with different IDH1 mutation state. **(B–J)** Methylation levels of promoter regions of the nine lncRNAs in glioma samples with different IDH1 mutation states. ^*^*p* < 0.05.

Supplementary Figure 2 Differential expression and prognosis of the five lncRNAs. **(A–E)** Boxplots show the relative expression levels of LICN00173 **(A)**, CAHM **(B)**, TMEM72-AS1 **(C)**, RAD21-AS1 **(D)**, and C1orf220 **(E)** in normal brain tissue and glioma. **(F–O)** The Kaplan-Meier curve for OS and DFS of glioma patients with LICN00173, CAHM, TMEM72-AS1, RAD21-AS1, and C1orf220 high expression and low expression using TCGA data. ^**^*p* < 0.01; ^***^*p* < 0.001.
