# Supplementary figures and images for: A Signature of Nine lncRNA Methylated Genes Predicts Survival in Patients With Glioma
Source: Front Oncol. 2021 Mar 22;11:646409. doi: 10.3389/fonc.2021.646409 (PMC8019920; doi:10.3389/fonc.2021.646409)

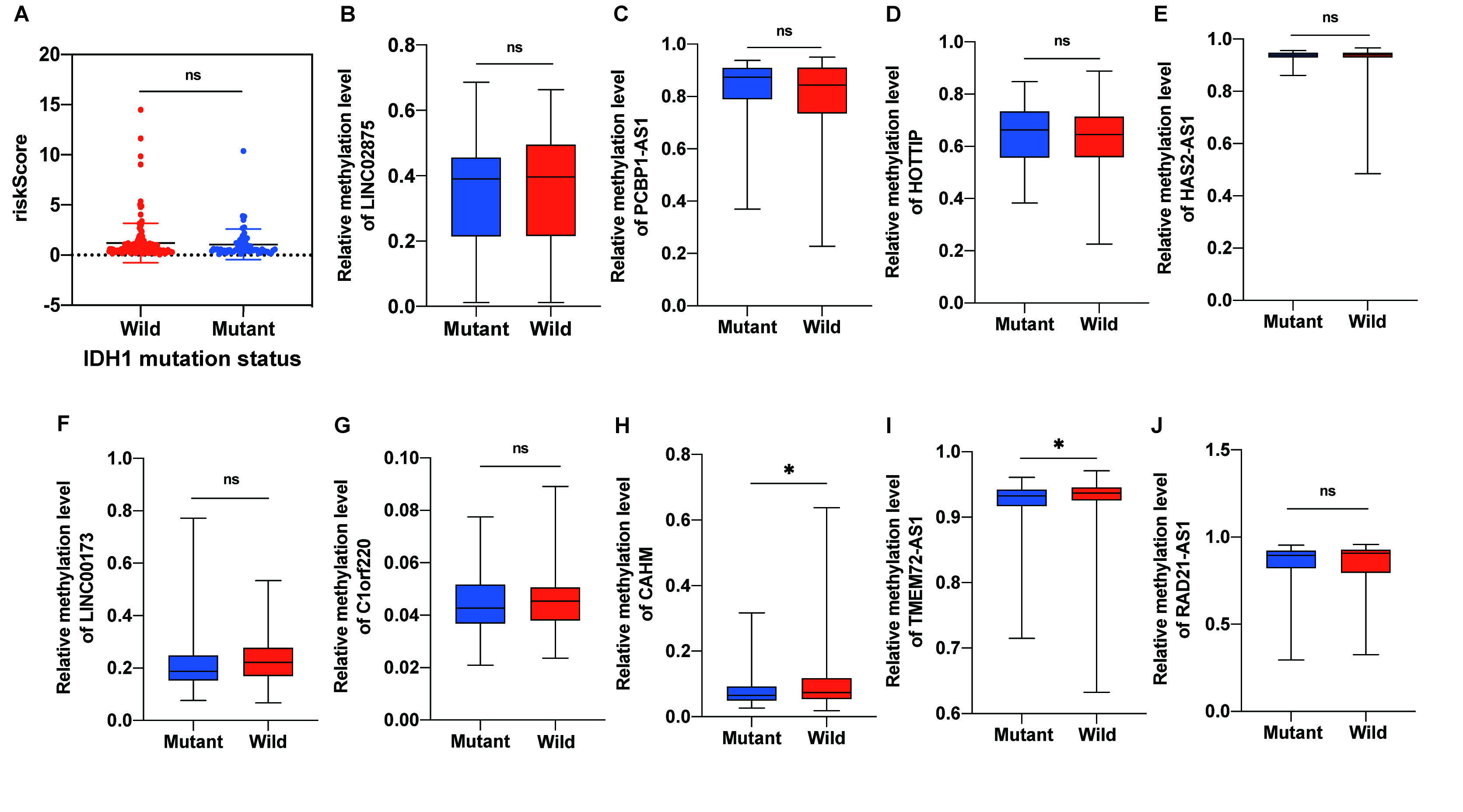

Supplement: Supplementary Figure 1 — (A) Boxplots show the risk assessment score of glioma patients with different IDH1 mutation state. (B–J) Methylation levels of promoter regions of the nine lncRNAs in glioma samples with different IDH1 mutation states. *p < 0.05. [file Image_1.TIF]

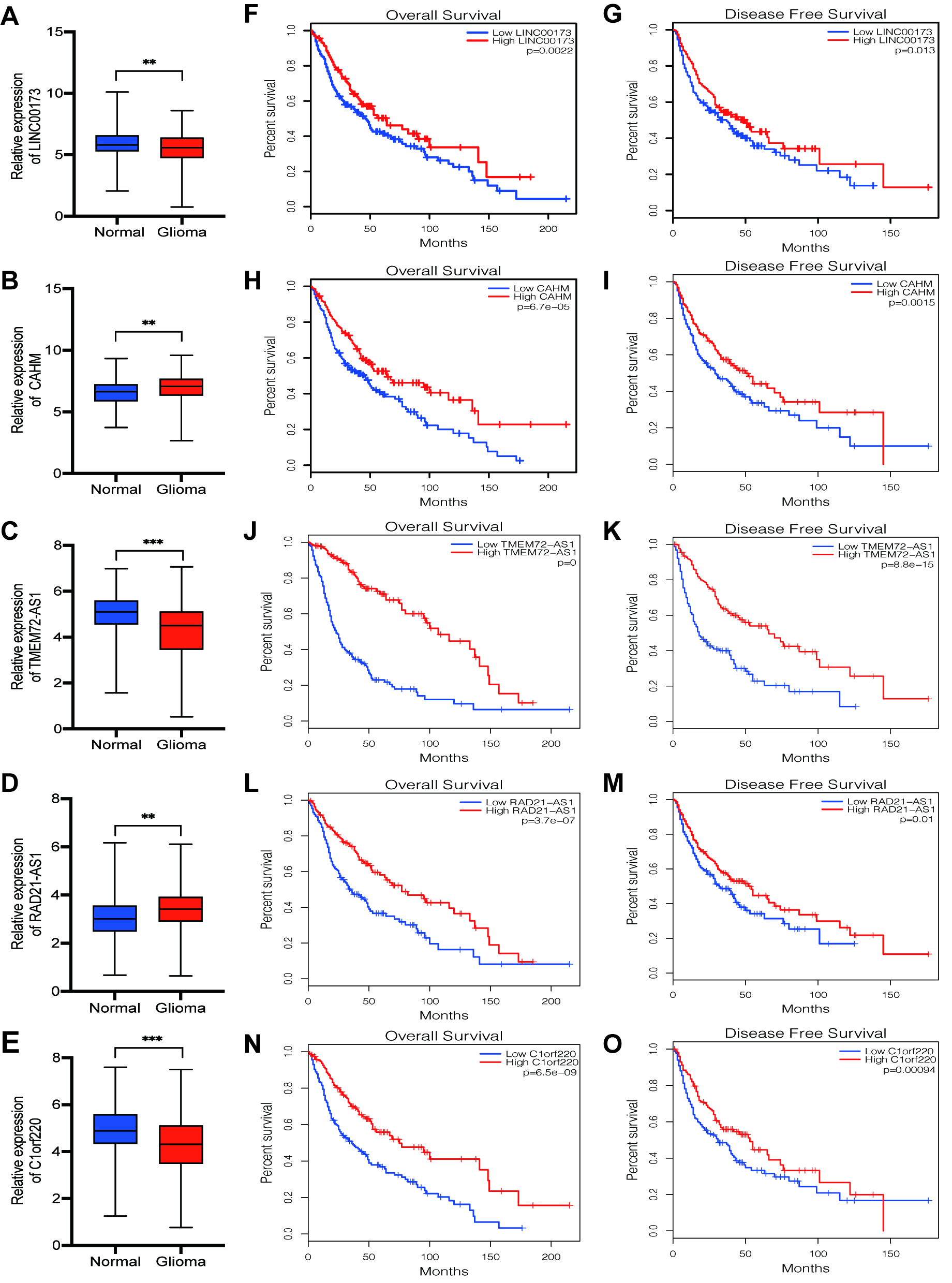

Supplement: Supplementary Figure 2 — Differential expression and prognosis of the five lncRNAs. (A–E) Boxplots show the relative expression levels of LICN00173 (A), CAHM (B), TMEM72-AS1 (C), RAD21-AS1 (D), and C1orf220 (E) in normal brain tissue and glioma. (F–O) The Kaplan-Meier curve for OS and DFS of glioma patients with LICN00173, CAHM, TMEM72-AS1, RAD21-AS1, and C1orf220 high expression and low expression using TCGA data. **p < 0.01; ***p < 0.001. [file Image_2.TIF]
